# Maternal dietary adherence during pregnancy to recommendations: a cross-sectional study in Modena

**DOI:** 10.1093/eurpub/ckac131.453

**Published:** 2022-10-25

**Authors:** L De Pasquale, L Palandri, MA Casalucci, D Azzalini, L Lucaccioni, E Passini, F Facchinetti, E Righi

**Affiliations:** 1 Department of Biomedical, Metabolic and Neural Sciences, University of Modena and Reggio Emilia, Modena, Italy; 2 Department of Medical and Surgical Science, University of Modena and Reggio Emilia, Modena, Italy

## Abstract

**Background:**

Unbalanced nutrients intake and incorrect weight gain can lead to immediate and future adverse health consequences for both mother and child. The Italian Society of Gynaecology and Obstetrics (SIGO), has drawn up a series of nutritional recommendations with the aim of promoting a correct food intake for future mothers. The purpose of our study was to assess adherence to good dietary indications during pregnancy and to evaluate if voluptuary habits could play a role.

**Methods:**

This cross-sectional study investigated dietary habits during the last trimester of pregnancy. We evaluated the adherence to dietary SIGO recommendations of a sample of pregnant women representative of physiologic full-term pregnancies (n = 572, mean age 33.4±5.2) living in Modena (Italy), recruited between 2016 and 2020. Maternal diet during pregnancy was assessed by a self-administered questionnaire fill in at the hospital after childbirth, evaluating lifestyle habits and usual food intake. Descriptive statistics and bivariate associations (Chi-square tests) were performed.

**Results:**

More than 50% of women did not comply with SIGO dietary recommendations. Overall, adherence was very low, ranging between 8.4% (sweets) and 38.8% (seafood), for all food categories, excluding coffee and tea (89%), alcohol (76.2%), red wine (99.1%) and seasoning (olive oil 93.4%). Preliminary results suggest that several factors and behaviours, including BMI before pregnancy, age, smoking habits, education, are associated with levels of adherence to different food categories.

**Conclusions:**

Poor adherence to a proper dietary regimen during pregnancy is a missed opportunity for prevention and demonstrates the importance of promoting public health interventions to improve dietary recommendations adherence. Several initiatives, such as courses, information campaigns, use of social media and counselling can be useful for a nutrition education in pregnancy, raising awareness of the related benefits for both mother and child.

**Key messages:**

• Nowadays pregnant women’s compliance to diet recommendations is still low.

• There is still a lot to do in terms of education and awareness of future mothers.

